# Long-Lasting Amelioration of Walking Trajectory in Neglect after Prismatic Adaptation

**DOI:** 10.3389/fnhum.2013.00382

**Published:** 2013-07-15

**Authors:** Marco Rabuffetti, Alessia Folegatti, Lucia Spinazzola, Raffaella Ricci, Maurizio Ferrarin, Anna Berti, Marco Neppi-Modona

**Affiliations:** ^1^Biomedical Technology Department, Fondazione Don Carlo Gnocchi ONLUS IRCCS, Milano, Italy; ^2^Department of Psychology, University of Torino, Torino, Italy; ^3^Department of Rehabilitation, Ospedale A. Bellini, Somma Lombardo, Italy

**Keywords:** neglect, rehabilitation, gait, prismatic adaptation, near space, far space, space representation

## Abstract

In the present study we explored the effect of prismatic adaptation (PA) applied to the upper right limb on the walking trajectory of a neglect patient with more severe neglect in far than in near space. The patient was asked to bisect a line fixed to the floor by walking across it before and after four sessions of PA distributed over a time frame of 67 days. Gait path was analyzed by means of an optoelectronic motion analysis system. The walking trajectory improved following PA and the result was maintained at follow-up, 15 months after treatment. The improvement was greater for the predicted bisection error (estimated on the basis of the trajectory extrapolated from the first walking step) than for the observed bisection error (measured at line bisection). These results show that PA may act on high level spatial representation of gait trajectory rather than on lower level sensory-motor gait components and suggest that PA may have a long-lasting rehabilitative effect on neglect patients showing a deviated walking trajectory.

## Introduction

Neglect patients behave as if the left part of the world had ceased to exist. As a consequence, both in clinical tasks and in many daily life activities, the patient’s behavior is usually biased toward the right side of space. It has also been demonstrated that neglect for proximal space (i.e., space within reaching distance) can be dissociated from neglect for distal space (space beyond reaching distance) (Halligan and Marshall, 1991; Cowey et al., 1994, 1999; Vuilleumier et al., 1998). In addition, near and far space representations were found to be dynamic, rather than static. Neurophysiological (Iriki et al., 1996) and neuropsychological studies (Berti and Frassinetti, 2000; Berti et al., 2002; Neppi-Mòdona et al., 2007) have shown that far space can me remapped as near, and near space as far, depending on the tool/action used by the patient to reach objects located in near and far space, respectively. Furthermore, among the functions that can be impaired in neglect there is walking, with patients showing a lateral deviation of the walking trajectory. Published research is contradictory regarding the direction of the lateral deviation, reporting both leftward and rightward deviations (Robertson et al., 1994; Tromp et al., 1995; Berti et al., 2002; Huitema et al., 2006; Turton et al., 2009). Leftward deviations have been found to be related to milder neglect (Tromp et al., 1995) or to a better preserved walking ability (Huitema et al., 2006).

Berti et al. (2002) have shown that neglect patients with more severe neglect in far than in near space produce a bisection error to the right (in the case of left neglect) of the true center of the line, when explicitly asked to walk across lines fixed to the floor in far space (3 m away). On the contrary, when the line was located in near space (1 m), the bisection error was less severe or even absent. This error pattern paralleled the bisection error made by the same patients in a line bisection task in near and far space using a projection light pen. Interestingly, patients’ walking trajectories were rectilinear when the line was located in far space. This suggested that the spatial representation activated at the beginning of the walking path (a far space representation, more severely impaired) was not updated during walking. Indeed, if this had been the case, a near (less impaired) space representation should have been activated while approaching the line: as a consequence, the trajectory would have been corrected resulting in a curvilinear path and the final error would have been reduced. The absence of spatial remapping during walking may be responsible for the collisions with objects and people occurring to neglect patients in their everyday life.

Although many different rehabilitative techniques have been effective in transitorily improving neglect, they often failed to produce a long-lasting beneficial effect. Some years ago, however, Rossetti et al. (1998) observed for the first time that wearing goggles fitted with prismatic lenses that shift the visual field 10°to the right may improve neglect in conventional neuropsychological tests performed in the patient’s peripersonal space. The positive result was already evident 5 min after prismatic adaptation (PA), lasting up to 2 h. Subsequent studies have shown that the effect of PA can be relatively long-lasting, being still effective up to 6 months post treatment (Frassinetti et al., 2002; Serino et al., 2006, 2007; Rusconi and Carelli, 2012).

In the present study we explored the effect of PA on the walking trajectory of a neglect patient with more severe neglect in far than in near space who was asked to repeatedly bisect a fixed line on the floor by walking across it. When neglect is more severe in far than in near space, two predictions can be made (Berti et al., 2002): (1) *space is not remapped*: the walking trajectory is rectilinear and the severity of neglect in far space determines the final bisection error; (2) *space is remapped:* the walking trajectory is curvilinear and the final bisection error is smaller because it is influenced by the near space representation (less compromised) activated while approaching the target. In both instances, if prism adaptation has a rehabilitative effect on the walking trajectory, it should produce a reduction of the final bisection error, either by improving the far space representation *at the beginning* of walking [in both cases 1 and 2), or by refining the remapping of far space into near space *during* walking (in case 2) only].

## Materials and Methods

### Patient’s clinical data

MR is a 56-year-old right-handed lady with 12 years of formal education. She worked as a teacher of primary school until her retirement at the age of 50. At the age of 55 she suffered from a subarachnoid hemorrhage, secondary to the rupture of a right posterior communicating artery aneurysm. She underwent a neurosurgical operation to evacuate the cerebral hematoma and the aneurysm was successfully clipped. However, after surgery she showed left hemianestesia, left hemiparesis, left hemianopia, and left visuo-spatial neglect. MR was severely impaired in daily life activities such as dressing, washing, and housekeeping. She obtained a low global score in the Activities of Daily Living (ADL score 10/20) (Wade, 1992).

Ten months after the stroke MR was considered for the present study while she was following both motor and cognitive rehabilitation training. Her walking ability had considerably improved, although she still reported difficulties in everyday life because of frequent collisions with obstacles located in her left space. She was still affected by left homonymous hemianopia, left hemianestesia, and chronic left neglect. Motor deficits were no longer detectable at the time of testing. We did not test MR for motor neglect. However, it may be inferred from the results of neuropsychological testing and from direct observation of her motor behavior that she did not suffer from motor neglect or directional hypokinesia (Bisiach et al., 1998): e.g., she bisected lines to the left of true center, a behavior opposite to that expected in case of directional hypokinesia, and had no problems and showed no reluctance in using her left arm for reaching objects, a behavior not compatible with motor neglect (see Saevarsson, 2013 for a critical review on diagnostic, clinical and anatomical issues related to premotor and motor neglect).

**Figure 1:**
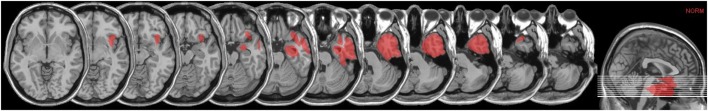
**Patient’s lesion reconstruction**. See text for details.

Lesion reconstruction from MRI scans showed a large lesion affecting the right temporal pole and extending, superiorly, to the Sylvian fissure and, posteriorly, to the more anterior temporo-medial structures, including the fusiform gyrus, the uncus, and probably, the amygdala (Broadman areas 38, anterior parts of areas 22, 21, 20, 36, 37) (see Figure 1). The patient gave her informed consent to participate in the study.

### Neuropsychological assessment

**Figure 2:**
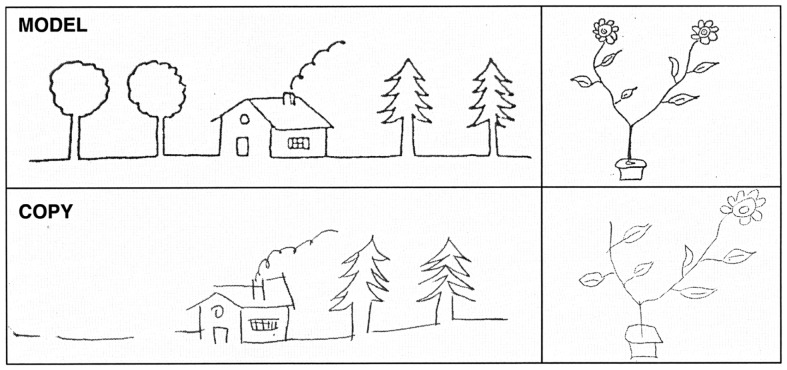
**Examples of patient’s copy of drawings**. Upper part of the figure: original; lower part: patient’s copy. Note that some details are missing on the left side of the copy.

When we evaluated MR, 10 months after the stroke, she was motivated and co-operative. Her performances in the Italian version of the Mini Mental State Examination (Measso et al., 1993) and in the Verbal Intelligence Judgment were normal (Spinnler and Tognoni, 1987). Her non-verbal intelligence performance (Carlesimo et al., 1995) was also normal. MR presented with severe *left* visual neglect as diagnosed on the basis of the performance on cancelation tests (Albert, 1973; Wilson et al., 1987) and drawing tests (Gainotti et al., 1972; Marshall and Halligan, 1993). Despite showing left neglect in these tasks (see Table 1 for details and Figure 2), she bisected line segments to the *left* of the objective midpoint (right neglect) both in conventional line bisection and in the walking bisection tasks. This behavior cannot be accounted for by hemianopia. In patients with neglect and hemianopia (such as MR), bisection errors are to the right of the objective midline (Doricchi and Angelelli, 1999; Doricchi et al., 2002). This kind of behavioral dissociation has been previously described in the literature (Berti et al., 2002) and will be further discussed in the Section “Discussion” (p. 14). The patient did not show personal neglect (Bisiach et al., 1986) or neglect dyslexia (Pizzamiglio et al., 1990).

**Table 1:** **General neuropsychological assessment**.

	Range	Cut-off	Score	Left omiss.	Right omiss.
**GENERAL COGNITIVE LEVEL**					
MMSE^1^	0–30	<23.8	25.99		
Verbal Judgment^2^	0–60	<32	60	
Raven’s Colored Progressive Matrices 47^3^	0–36	<18.96	21	8/12	0/12
**NEGLECT (CONVENTIONAL TESTS)**					
Albert’s test^4^	0–50	>1 omission	45^*^	4/25	1/25
Star cancelation^5^	0–54	>3 omission	22^*^	27/27	5/27
Word reading^6^	0–40	1	40		
Sentence reading^7^	0–9	1	9		
Personal neglect^8^	0–3	≤1	0		

^*^Pathological Score; ^1^Measso et al. (1993); ^2^Spinnler and Tognoni (1987); ^3^Carlesimo et al. (1995); ^4^Albert (1973); ^5^Wilson et al. (1987); ^6^Caramazza and Hillis (1990); ^7^Pizzamiglio et al. (1990); ^8^Bisiach et al. (1986).

### Experimental procedure

The experimental procedure for the detection and characterization of bisection errors included several manual and walking bisection tests and was applied before and after each of four sessions of PA (see Prismatic Adaptation below). Moreover, bisection errors were also measured in two follow-up sessions.

#### Manual line bisection

In order to assess the presence of dissociations between neglect in near and far space (Halligan and Marshall, 1991; Cowey et al., 1994, 1999) patient MR was asked to bisect line segments made of 30 mm large white tape fixed to the floor. In *near space* the target line was located at a distance of 0.75 m from the patient’s feet and she had to bisect the line by reaching it with a carbon fiber stick. In *far space* the target line was located 3 m from the patient’s feet and the patient bisected the line by means of a laser pointer. The two conditions *reaching in near space* and *pointing in far space* were considered the “baseline” conditions to reveal the presence of dissociations between neglect in near and in far space (see Berti and Frassinetti, 2000; Pegna et al., 2001; Neppi-Mòdona et al., 2007). When using a stick to bisect a segment located in near space or a laser pointer to bisect a segment located in far space, patients do not remap near space into far space or far space into near space, respectively; instead, when using a laser pointer to bisect a segment located in near space, an object-dependent far space representation can be activated (a laser pointer is often associated to actions carried out in far space); similarly, when using a stick to bisect a segment located in fare space, a near space representation can be activated (the stick activates a near space representation because the far object, once reached with the stick, is automatically recoded as being located in proximal space as a consequence of tool use). A patient is considered to have a dissociation if the bisection errors in near and far space are significantly different.

The target lines were centered on the patient’s body midline. MR executed a total of 20 bisections (10 in near and 10 in far space). The length of the line was varied in near and far space so as to keep the visual angle subtended by each line constant (24.5°). Lines in near space were 0.71 m long whereas lines in far space were 1.45 m long. Bisection errors were measured as deviation in mm from the objective midpoint of the line and expressed as percentage of target line half-length (NBE, Near space Bisection Error; FBE, Far space Bisection Error). Positive values indicate deviations to the right of the objective midpoint, whereas negative values indicate deviations to the left.

#### Line bisection by walking

The patient was also asked to bisect lines in near and far space by walking across them. This allowed us to assess whether a possible dissociation between near and far space neglect was consistent across different output modalities (manual/walking bisection). The lines were identical to those used in the bisection by reaching/pointing and were placed at a distance of 0.75 m (near space) and 3 m (far space) from the patient’s starting location. She was instructed to cross the line in the middle, taking her body midline as reference point. We did not advise the patient to walk as straight as possible because this instruction could interfere with the task and influence the results of the experiment (for example it could interfere with spatial remapping during gait execution by driving the patients attention to her walking rather than to the bisection task itself). As for bisection by reaching/pointing, she was given a sequence of 10 trials for each spatial sector, for a total of 20 trials (10 in near and 10 in far space). No environmental cues were available to the patient to guide her walking trajectory (a large uniform light green carpet completely covered the floor and a 5-m wide uniform cyan curtain was hanged about 2 m behind the target line).

The measurement of the trajectories was performed by means of an ELITE optoelectronic motion analysis system (BTS, Milan, Italy) whose sensors consisted in four TV cameras working in the infrared range and focused on a calibrated volume (length: 5.0 m; height: 1.7 m; width: 1.2 m) intended to include the subject, the starting point and the target line. Three passive hemispherical reflective markers (diameter: 15 mm) were placed on the patient’s body in correspondence of specific anatomical landmarks: the sacrum and the posterior aspect of the calcaneus on both feet, while two markers were placed at both line extremities and one on the starting point. The patient was dressed normally and wore her regular walking shoes. The TV cameras recorded the marker trajectories at a sampling frequency of 100 Hz. Specific stereophotogrammetric algorithms made it possible to compute the 3D instant position of any marker detected by at least two TV cameras. Such setup and related algorithms provide an accuracy that is approximately 1/3000 of the calibrated volume’s largest dimension, therefore the experimental accuracy of the measurements was about 2 mm. Raw coordinates data were low pass filtered (cut-off frequency 2 Hz). The sacrum was assumed as the body reference point, being strongly correlated with the body center of mass during walking (Thirunarayan et al., 1996). The sacrum trajectory actually consists of different components:

- a major rectilinear progression component;
- a possible curvilinear component which accounts for possible walking steering;
- small cyclic lateral and vertical oscillations due to the particular mechanics of bipedal walking (Inman et al., 1981).

**Figure 3:**
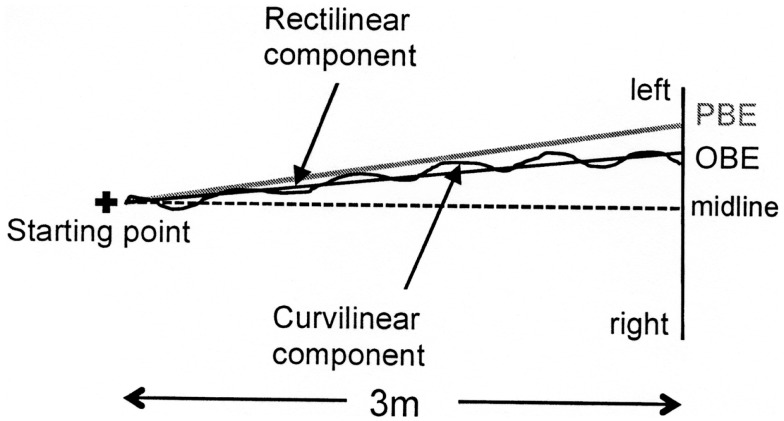
**Graphic representation of a typical walking trajectory of patient MR and its components: (1) a major rectilinear component and (2) a curvilinear component forming a second-order polynomial curve (the progression curve) whose intersection point with the target line corresponds to the observed bisection error (OBE)**. The point of intersection with the target line of the tangent to the “progression curve” at the starting point identifies the bisection error predicted by the first walking step (PBE).

The latter component is not relevant for the current study. A geometrical model of the first two components, the “progression curve,” was defined in order to identify a second-order polynomial curve (Y = aX^2^ + bX + c), where the instant lateral displacement is a function of the longitudinal component (see example in Figure 3). The elements of this second-order polynomial curve include the major rectilinear direction (first order component) and the possible veering (second order component).

Two bisection error parameters were computed from all identified progression curves (see Figure 3). The first error was Observed Bisection Error (OBE): the actual bisection error measured at the end of the walking trajectory (intersection of the progression curve with the target line). The second error was Predicted Bisection Error (PBE): the bisection error predicted on the basis of the initial walking direction (point of intersection with the target line of the tangent to the “progression curve” at the starting point). Both OBE and PBE are expressed as percentages of the target line half-length and can be preceded by a positive or a negative sign indicating errors to the right and to the left of the target line midpoint, respectively.

**Figure 4:**
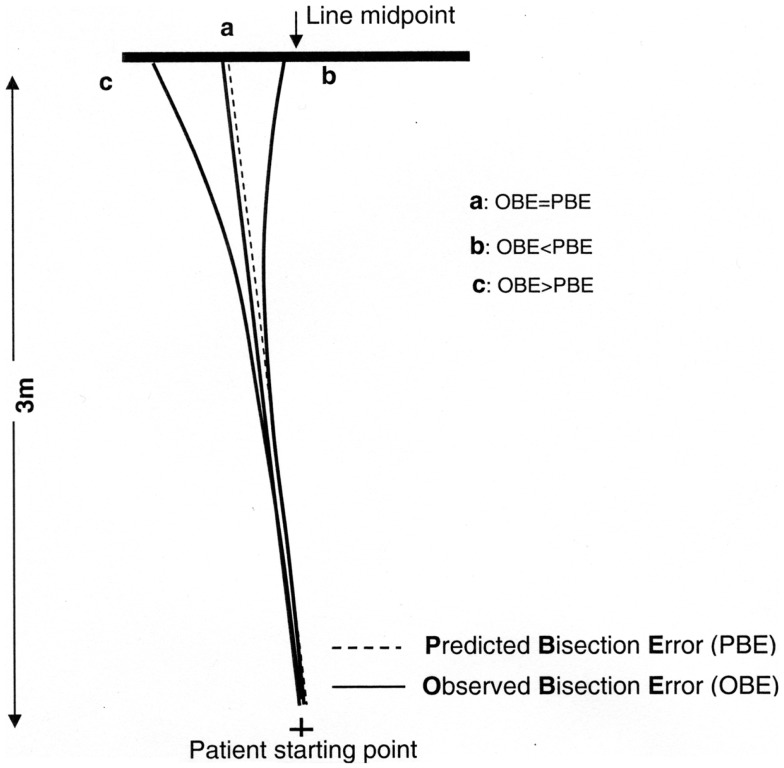
**Graphic representation of different possible walking trajectories of the patient and observed (OBE) or predicted (PBE) bisection errors: if OBE = PBE** (a) **the trajectory is rectilinear and space remapping is absent**. The far space representation (more compromised) activated at the beginning of walking is maintained throughout the entire path, resulting in a leftward error; if OBE < PBE (b) the trajectory is curvilinear because the patient corrects the trajectory while approaching the target remapping the initial far space representation (more compromised) into a near space representation (less compromised). This results in an error reduction relative to (a); if OBE > PBE (c) it is indicative of a tendency to veer in the direction of neglect during walking not related to space remapping but consequent to an error in heading control resulting from the patient attempt to correct the ipsilesional deviation of the subjective midline which is typically associated to neglect (Huitema et al., 2006).

If OBE = PBE, the walking trajectory is rectilinear, indicating that the patient did not change gait direction while walking (Figure 4, case a). Conversely, if OBE ≠ PBE, gait direction has changed according to a curvilinear trajectory, indicating that spatial remapping has occurred. If the absolute value of OBE is lower than the absolute value of PBE (|OBE| < |PBE|) and both errors are toward the same side of the target line, the patient has corrected the initial trajectory progressively reducing the bisection error while approaching the line (Figure 4, case b). Therefore, the difference (|PBE| − |OBE|) can be considered an index related to the curvature of the walking trajectory and to the occurrence of remapping.

We could also consider the unexpected, but nonetheless theoretically possible, condition that |OBE| > |PBE|. In this case the correction of the patient’s trajectory would not be the consequence of spatial remapping, but would be the result of a defective heading control while walking (Figure 4, case c). Huitema et al. (2006) suggested that neglect patients with a preserved walking ability – as is the case for patient MR – when asked to walk toward a target might veer toward the left as a consequence of an attempt to compensate for the rightward deviation of their subjective midline.

#### Prismatic adaptation

In order to improve neglect for left space, patients can be treated with wedge prisms shifting the visual field to the ipsilesional *right* side while performing pointing movements with the ipsilesional hand toward a visual target located in near space. The rightward optical deviation initially causes an ipsilesional pointing error, i.e., patients misreach the targets to the right of their actual position (pre-adaptation error). After a variable number of trials, they spontaneously correct the visual shift induced by the prisms by directing their pointing movements to the contralesional (left) side until they aim correctly for the target (adaptation effect). Once prisms are removed, patients show a directional pointing error toward the contralesional *left* side (after-effect).

Patient MR, despite having suffered a lesion to the right hemisphere, bisected lines to the *left* of the objective midpoint and veered to the left while walking. The presence of this leftward bias made us consider the opportunity to orient the wedge prisms so as to deviate the visual field to the *left* in order to obtain a realignment of visuo-motor coordinates to the right (after-effect). However, given that MR’s left neglect was still apparent in copying and cancelation tasks, we decided to apply the adaptation procedure normally employed with left neglect patients using prismatic lenses deviating the visual field to the *right*. Therefore, MR wore a pair of prismatic goggles fitted with wide-field point-to-point 20 diopters lenses that induced a 10°*rightward* optical deviation. During the PA procedure, she was asked to repeatedly point with her right index finger, with a one shot movement, to four small black filled circles (1 cm in diameter and numbered 1–4), horizontally aligned, and centered on the vertical axis of an a A3 sheet of paper. The A3 sheet of paper was centered on the patient’s midsagittal plane and was located at a distance of 50 cm. PA involved a total of 120 randomized pointing movements grouped in three sequences of 40 movements each, and required approximately 20 min to be completed. Upon verbal command of the examiner, the patient pointed at one of the four numbered circles while wearing a lattice glove. Her right index finger was inked so as to leave a visible mark on the sheet. For each pointing movement, a pointing error was measured to the nearest mm (i.e., the lateral displacement of the center of the mark from the target).

The patient received four sessions of PA, distributed over a time span of 67 days: the second session was administered 1 week after the first session, while there was a 1 month interval between the second and third and the third and fourth session. In order to evaluate the presence of long-lasting effects of PA on bisection performance, two follow-up sessions were conducted 3 months and 15 months after the last training session (session 4).

### Predictions

**Figure 5:**
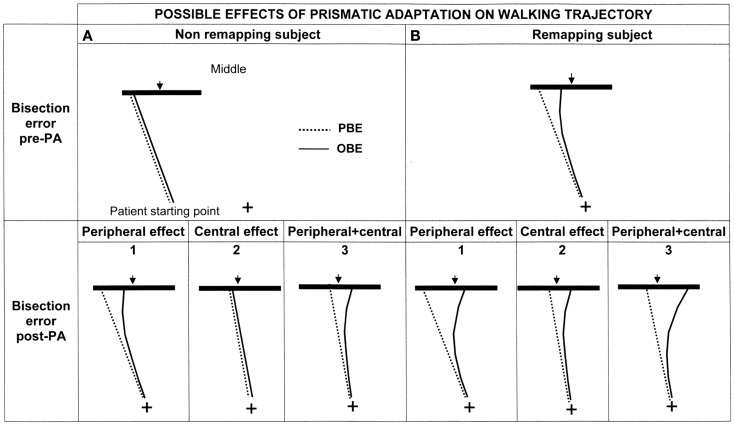
**Graphic representation of the possible effects of PA on the walking trajectory of patient MR depending on the presence (B: upper right panel) or absence (A: upper left panel) of spatial remapping and on the level at which PA is effective (lower panels: 1 = peripheral effect; 2 = central effect; 3 = peripheral + central effect)**. See text for details.

Predictions need to take into account two factors: presence/absence of space remapping and the nature (peripheral/central) of the effect of PA (see Figure 5). Indeed, normal human behavior implies a rectilinear walking direction with null PBE and OBE. This instance is not included in the figure, where it is only considered the pathological behavior showing a deviated walking trajectory (|PBE| > 0).

#### Space remapping

Considering that patient MR showed more severe neglect in far rather than in near space – see Section “Results” – we may advance two hypotheses (Berti et al., 2002) in relation to space remapping that make different predictions regarding the bisection performance in the walking modality: (1) *space is not remapped* during walking (Figure 5A, upper section). In this case, because space representation is not updated during walking, the trajectory is assumed to be rectilinear and the *first representation* that is activated (the representation of far space, in our patient the most impaired one) should be the one responsible for the bisection performance. In this case OBE = PBE or, alternatively, OBE is not significantly different from PBE; (2) *space is remapped* during walking. In this case, patient MR should activate the most impaired representation at the beginning of each walking path and the less impaired, or even unimpaired, representation toward the end. Her walking trajectories should, therefore, be deviated at the beginning of each walking path, when the starting point is at 3 m, and then, gradually, as she approaches the line, with the activation of the more preserved representation, they should be corrected. According to this hypothesis the *last representation* that is activated should be the one responsible for the line bisection performance. Because this prediction implies a correction of the trajectory during walking, OBE should differ from PBE, in particular |OBE| < |PBE| (Figure 5B, upper section).

#### Effect of prismatic adaptation

If PA is effective in improving the patient’s walking trajectory, we expect to see a reduction of bisection errors. Three hypotheses may be advanced, for both remapping or not remapping subjects, in relation to the processing level at which PA is effective (see Figure 5, lower panel):

1. PA mainly acts at a peripheral level by realigning the visuo-motor coordinates *during walking*. In this case the effect should be evident on OBE and not on PBE, because trajectory correction should manifest during walking rather than from the first step (pre-treatment PBE will be equal to post-treatment PBE, whereas post-treatment OBE will diminish: hence, the curvature of the trajectory post treatment will increase if already occurring before PA (Figure 5B1) or be newly introduced if absent before PA (Figure 5A1).
2. PA acts at a higher level (at the level of space representation). In this case the correction should be evident at the beginning of walking and affect PBE, because it would be due to a restoring of the functioning of far space representation *before* the initiation of walking. Post-treatment PBE will be smaller, i.e., less deviated, than pre-treatment PBE: as a consequence, also OBE will decrease of a substantially equivalent amount and the curvature of the trajectories pre and post treatment will be substantially the same, depending on the presence (Figure 5B2) or absence of remapping (Figure 5A2).
3. PA acts at both levels. In this case its effect is a combination of the effects previously predicted and, therefore, PBE and OBE should change at the beginning of walking (effect on space representation) and during walking (effect on space remapping) (see Figures 5A3,B3).

## Results

In the following analyses, the dependent variables (bisection errors in reaching/pointing tasks and in walking tasks computed prior to each PA session) are expressed as % deviation with respect to half line length – positive values indicate a rightward error, negative values indicate a leftward error. In order to investigate the presence of dissociations between near and far space neglect, we evaluated bisection errors both in near and in far space. The effect of PA, instead, was evaluated in far space only. The reason is twofold: (1) neglect was absent in the manual bisection task in near space (the bisection error (−3.0%) was not significantly different from the null value in a One Sample *t* test: *t*_9_ = −1.30; *p* = 0.23); (2) in the walking bisection task it is possible to investigate the occurrence of spatial remapping of gait trajectory only when the line is located in far space. A summary of the results is reported in the subsequent Tables 2 and 3.

**Table 2:** **Bisection by pointing in far space (FBE)**.

FBE	S1	S2	S3	S4	Follow-up 1
Pre PA	−13.1 (7.7)	−15.4 (6.6)	−10.5 (9.7)	−0.8 (11.0)	−21.6 (8.8)
Post PA	−3.0 (11.4)	−16.1 (11.3)	4.2 (5.8)	−8.6 (5.2)	

**Table 3:** **Bisection by walking in far space**.

	S1	S2	S3	S4	Follow-up 1	Follow-up 2
**PBE**					
Pre PA	−54.3 (15.8)	−37.8 (39.0)	−43.7 (39.0)	−24.4 (26.0)	−28.5 (20.1)	−29.3 (15.6)
Post PA	−51.8 (10.3)	−24.0 (25.5)	−20.5 (23.3)	−11.0 (36.2)		
**OBE**					
Pre PA	−8.2 (13.3)	−9.1 (9.0)	−2.1 (10.6)	−2.7 (9.3)	−4.1 (9.6)	0.5 (4.4)
Post PA	1.8 (7.3)	−6.6 (15.9)	5.9 (6.1)	3.8 (6.6)		

### Dissociation between far and near space neglect in the manual bisection task

**Figure 6:**
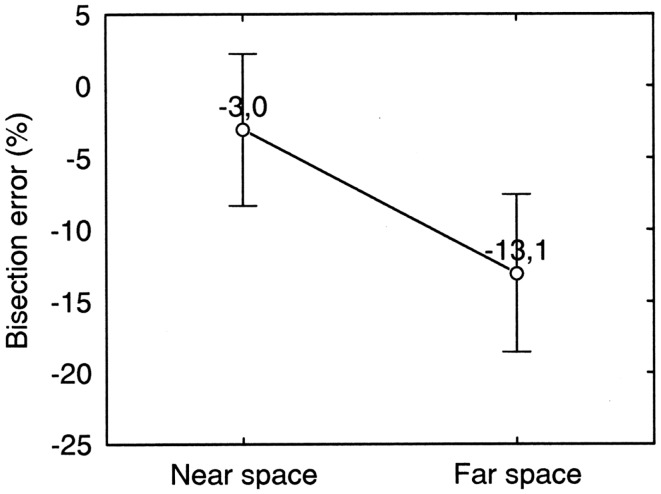
**Near and far space bisection errors of patient MR in the manual bisection task**. Error bars represent standard errors. Bisection error is expressed as % deviation with respect to half line length. Negative and positive values indicate errors to the left and to the right of the objective midline, respectively. The error is to the left of the true center of the line and is significantly larger in far space than in near space.

Mean errors in bisection tests performed with a stick in near space and with a laser pointer in far space (baseline conditions), are presented in Figure 6.

Each point represents average data from the first pre-treatment session. As evident from Figure 6, MR bisected lines to the left of the objective midpoint. This behavior is usually associated to right-sided neglect. However, MR had a right brain lesion and left sided neglect in copying and cancelation tasks. (See section Discussion for a discussion of this point). Moreover, a strong dissociation between near and far space neglect was present. There was significantly more bisection error in far space (−13.1%) than in near space (−3.0%) (Paired Samples *t* test: *t*_9_ = 2.63; *p* = 0.03) and the latter was not significantly different from 0 (One Sample *t* test: *t*_9_ = 1.30; *p* = 0.23).

### Dissociation between far and near space neglect in the bisection by walking tasks

**Figure 7:**
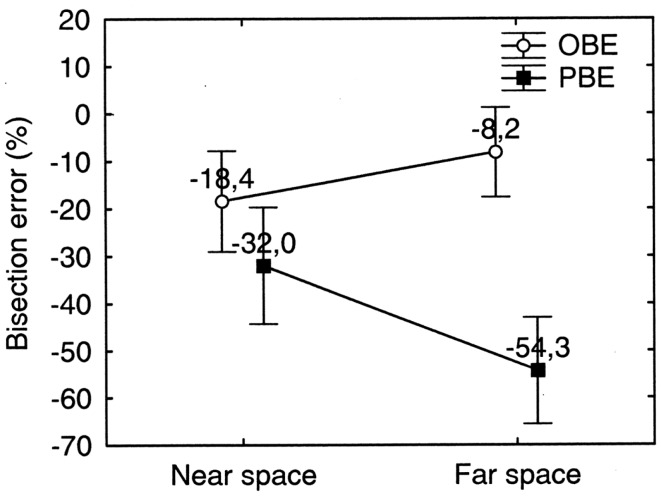
**Far and near space bisection errors of patient MR in the walking bisection task**. Error bars represent standard errors. Bisection error is expressed as % deviation with respect to half line length. OBE, observed bisection error; PBE, predicted bisection error. Negative and positive values indicate errors to the left and to the right of the objective midline, respectively. Neglect is more severe in far space than in near space: PBE in far space is significantly greater than OBE in near space. See text for details.

Similarly to the manual bisection condition, a (weak) dissociation between far and near space neglect was found in the bisection by walking condition. Indeed, in far space, neglect was significantly worse than in near space when we compare PBE in far space with OBE in near space [PBE_far_ (−54.3%) vs. OBE_near_ (−18.4%): *p* = 0.0002 on Newman–Keuls *post hoc* test] (see Figure 7). These two error parameters can be considered the baseline conditions to assess the presence of dissociations between neglect in near and in far space because both are free from any effect related to spatial remapping during walking. A repeated measures ANOVA with ERROR PARAMETER (pbe/obe) and SPACE (Near/Far) as two levels within subjects factors and % bisection error as dependent variable showed a significant main effect of ERROR PARAMETER (*F*_1_ = 24.68, *p* < 0.001) and of the interaction between the two factors [*F*_(1, 9)_ = 36.04, *p* < 0.001]. The main effect of ERROR PARAMETER indicates that the patient corrected her trajectories during walking, as evidenced by the fact that average OBE was smaller (−13.3%) than average PBE (−43.17%). OBE, however, remained significantly>0% on a One sample *t* test (*t*_9_ = −3.93; *p* = 0.003). The significant effect of the interaction ERROR PARAMETER ^∗^SPACE apparently suggests that PBE and OBE dissociate in far and near space (PBE appears more severe in far space whilst OBE appears more severe in near space). However, this interpretation is incorrect and should be reconsidered taking into account the fact that the reduction of OBE from a near to a far starting location is determined by spatial remapping of far space (more compromised) into near space (less compromised) while approaching the target from a far starting location. The error reduction is smaller when the starting location is in near space (0.75 m from target) probably because the near space representation activated at the beginning of the walking path needs more than a single footstep to induce a reduction of the bisection error comparable to that observed when the starting location is in far space.

### Prismatic adaptation

**Figure 8:**
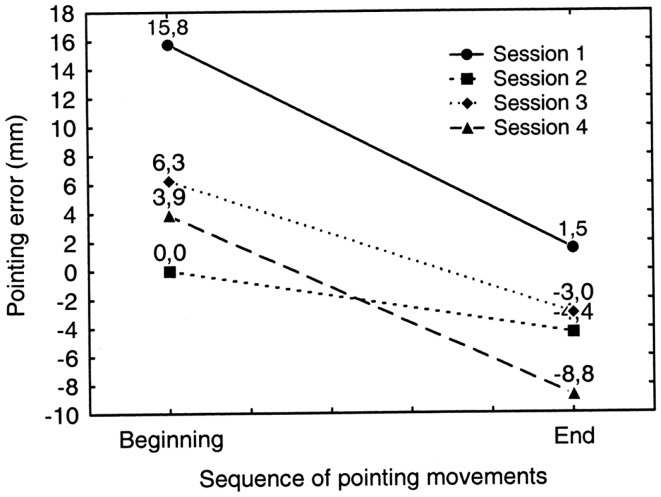
**Graphic representation of the occurrence of prismatic adaptation (PA) in patient MR during each treatment session (session 1–4)**. Positive and negative values indicate deviations to the right and to the left of the target, respectively. Adaptation occurs if the rightward pointing error measured at the end of the adaptation procedure (End: pointing movements no. 113–120) is significantly smaller than the error measured at the beginning of the adaptation procedure (Beginning: pointing movements no. 1–8). Error reduction is significant in every treatment session except in session no. 2. See text for details.

In order to assess the occurrence of PA we compared the average error at the beginning of the adaptation phase (initial sequence of eight pointing movements: no. 1–8) with the average error at the end of the adaptation phase (final sequence of 8 pointing movements: no. 113–120) of each treatment session (four sessions) (see Figure 8). A repeated measures ANOVA was performed on the pointing error on the horizontal plane measured in mm (dependent variable) as a function of PA phase (initial sequence of pointing movements/final sequence of pointing movements) and of PA session (1–4) as within subjects factors. Both factors resulted statistically significant [PA phase: *F*_(1, 7)_ = 15.73; *p* < 0.005; PA session: *F*_(3, 21)_ = 11.41; *p* < 0.001]. In all the sessions, except the second one, the pointing error reduction at the end of the adaptation phase was significant (*p* < 0.05 for all comparisons at paired samples *t* tests, two tailed; Error reduction: session 1 = 14 mm; session 2 = 4 mm; session 3 = 9 mm; session 4 = 13 mm). This indicates that the patient consistently adapted to the optical shift induced by prisms. The main effect of the variable session, it has to be ascribed to the significantly greater pre-adaptation mean error in the first PA session than in all of the following sessions. In fact the mean pre-adaptation error in the first PA session (16 mm) was significantly greater than the pre-adaptation error measured in session 2 (0 mm), session 3 (6 mm), and session 4 (4 mm) (all comparisons are significant at paired samples *t* tests, one tailed, *p* < 0.01). The pre-adaptation error reduction in sessions 2 through 4 is due to the fact that the massive adaptation obtained in the first session is substantially maintained in the subsequent sessions: indeed, the mean post-adaptation error in session 1 was comparable to the pre-adaptation error in sessions 2, 3, and 4 (all comparisons *p* > 0.3 at paired samples *t* test, two tailed) (See Figure 8).

### Effect of prismatic adaptation in the bisection by pointing task

**Figure 9:**
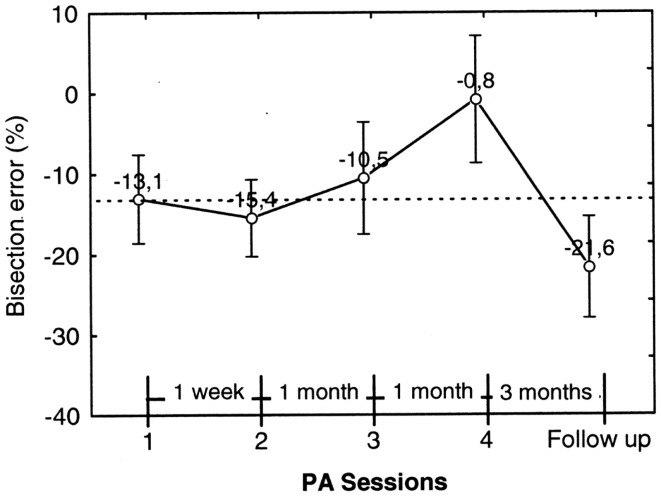
**Graphic representation of the effect of prismatic adaptation (PA) on the patient’s bisection by pointing errors in far space**. Each data point represents the bisection error prior to PA. The error is significantly reduced in session 4. However, at follow-up (3 months after session 4) the effect of PA is no longer present and neglect is as severe as prior to PA. Error bars represent standard errors. Bisection error is expressed as% deviation with respect to half line length. Negative and positive values indicate errors to the left and to the right of the objective midline, respectively.

Figure 9 shows the trend of the bisection error in far space prior to each PA session (1–4) and at the follow-up 3 months after session 4. It is to be noted that, starting from session 2, the pre-adaptation bisection error incorporates the effect (if any) of PA of the preceding session.

In order to test statistically the effect of PA sessions on neglect, we ran a repeated measures ANOVA on bisection error prior to PA (dependent variable) as a function of PA sessions (within subject factor, four levels: PA session 1–4). PA session resulted significant [*F*_3, 27=_5.83; *p* = 0.003]. Neglect significantly improved in session 4, where bisection error was close to 0% and was significantly less severe than in sessions 1–3 (*p* ≤ 0.01 at Newman–Keuls *post hoc* for all comparisons). However, neglect reappeared in the follow-up session, which occurred 3 months after session 4, and was significantly worse than in session 1, 3 and 4 (*p* = 0.01, *p* = 0.03, and *p* < 0.01, respectively, at Paired samples *t* tests). For this reason we did not run a second follow-up.

In summary, the bisection error in far space not only was significantly reduced by PA, but disappeared after three sessions of treatment carried out over a period of 37 days; this improvement was still evident a month later (session 4). However, at the follow-up 90 days after session 4, neglect reappeared and was comparable to neglect prior to treatment.

### Effect of PA in the bisection by walking tasks

**Figure 10:**
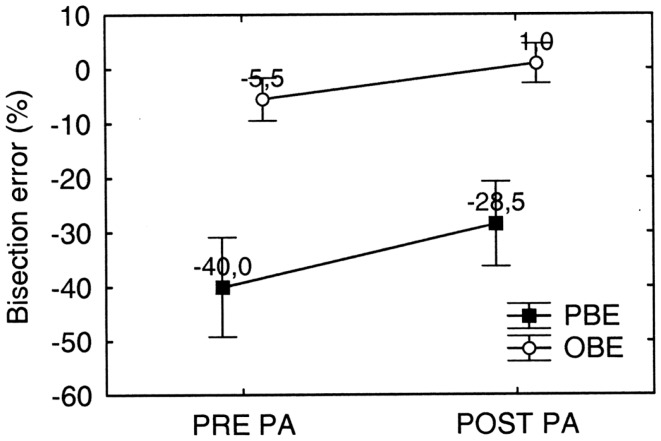
**Graphic representation of the overall effect of prismatic adaptation (PA) on MR’s bisection by walking error in far space**. PA is effective in significantly reducing both the observed (OBE) and the predicted (PBE) bisection error. PBE is significantly larger than OBE, but error reduction due to PA is comparable. Error bars represent standard errors. Bisection error is expressed as % deviation with respect to half line length. Negative and positive values indicate errors to the left and to the right of the objective midline, respectively. See text for details.

The overall effect of PA on bisection error in the walking tasks is shown in Figure 10, where each point represents the average bisection error of four PA sessions collapsed together. Consistently with Figure 10 the direction of the bisection error already observed in the preliminary bisection test (see Figure 7), the patient crossed the line to the left of its true center, showing apparent right-sided neglect.

**Figure 11:**
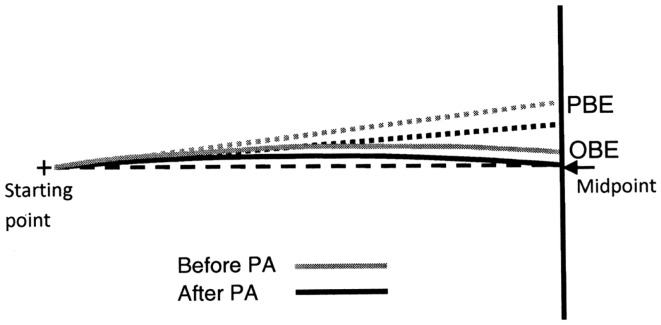
**Graphic representation of the shape of MR’s predicted (dotted lines) and observed (continuous lines) walking trajectories prior (black lines) and after (gray lines) prismatic adaptation (PA)**. Note that PA reduces both the predicted (PBE) and the observed (OBE) bisection error but does not modify the shape of the trajectories (continuous lines), showing that spatial remapping in patient MR is independent of PA.

In order to test statistically the overall effect of PA on walking tasks, we performed a repeated measures ANOVA on bisection error (dependent variable) as a function of PA (two levels: Pre PA/Post PA) and Error Parameter (two levels: PBE/OBE) as within subjects factors. Both factors significantly influenced bisection performance [PA: *F*_(1, 9)_ = 12.29; *p* < 0.001; Error Parameter: *F*_(1, 9)_ = 115.67; *p* < 0.0001], while the interaction PA^∗^ Error Parameter was not significant. The main effect of Error Parameter showed that the bisection error predicted at the beginning of the walking trajectory (PBE = −40.0%) was more severe than the bisection error observed at the end of the walking trajectory (OBE = −5.5%), indicating that the patient remapped the representation of far space – more compromised – into near space – more preserved – while approaching the target (see also Figure 11). Moreover, the significant effect of PA shows that it was effective in reducing neglect.

Interestingly, PA significantly reduced both PBE (PBE_post PA_-PBE_pre PA_ = −11.5%: *p* < 0.001 on Newmann Keuls *post hoc*) and OBE (OBE_post PA_ − OBE_pre PA_ = −6.5%: *p* = 0.037 on Newmann Keuls *post hoc*) (see Figure 10). Despite the effect of PA on OBE was (non-significantly) smaller (*p* = 0.21 on Paired samples *t* test), the difference between PBE and OBE prior and after treatment was comparable [(PBE-OBE)_Pre_ = −34.5%; (PBE-OBE) _Post_ = −29.4%; *t*_9_ = −1.33: *p* = 0.21 on paired samples *t* test]. Since the difference (PBE-OBE) quantifies the effect of gait direction changes due to spatial remapping, this result indicates that PA had no significant effect on the shape of the trajectories, which already showed the effect of spatial remapping before PA (see also Figure 11 for a graphical representation).

**Figure 12:**
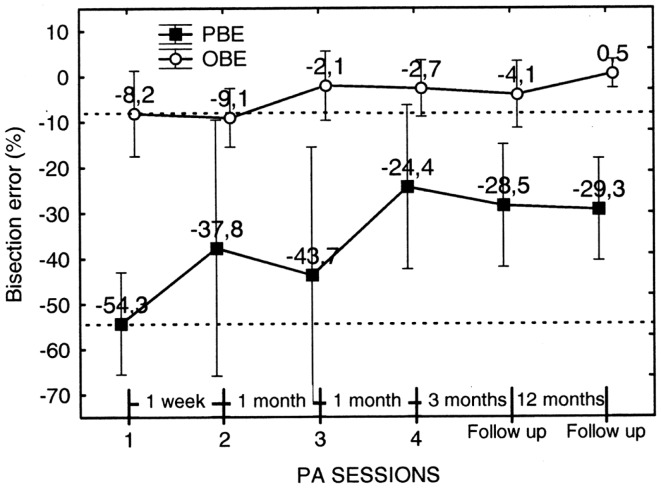
**Graphic representation of the effect on the walking bisection error of individual PA sessions (1–4) and of the duration of the effect (follow-up)**. Each point represents the pre-adaptation bisection error, i.e., the error measured prior to each PA. The pre-adaptation error measured in session 1 serves as baseline (upper and lower dashed lines). Note that the effect of PA in terms of error reduction a) is greater for the predicted (PBE) than for the observed (OBE) bisection error and b) it is maintained at follow-up 3 and 15 months after the last PA session (no. 4). Error bars represent standard errors. Bisection error is expressed as % deviation with respect to half line length. Negative and positive values indicate errors to the left and to the right of the target objective midline, respectively. See text for details.

In order to analyze the effect of each PA session on bisection error, we performed an additional repeated measures ANOVA on pre-adaptation bisection error as a function of PA session (four levels: sessions 1–4) and Error parameter (two levels: PBE and OBE) as within subjects factors (see Figure 12). Consider that the pre-adaptation bisection error measured on session 1 is free from any effect of treatment and it can be considered as the baseline condition. Starting from session 2, instead, the pre-adaptation bisection error incorporates the effect (if any) of PA of the preceding session.

The analysis showed that the factor Error Parameter was, indeed, significant [*F*_1, 9_ = 8.43; *p* < 0.0001], with the overall error predicted at the beginning of the walking trajectories resulting more severe than the error observed at the end of the walking trajectories. The factor PA Session, instead, resulted non-significant. However, PBE measured in session 4 resulted significantly smaller than PBE in session 1 (*p* = 0.02 on paired samples *t* test). Furthermore, PBE measured at follow-up 3 and 15 months after session 4 remained significantly smaller than in session 1 (*p* = 0.016 and *p* = 0.007, respectively, on paired samples *t* tests) and was comparable to the error measured in session 4. Considering OBE, the effect of PA session was of smaller entity. We compared OBE of session 1 and 2 collapsed together (mean error = −8.65%) with OBE of follow-up sessions 1 and 2 collapsed together (mean error = −1.83%) (we collapsed session 1 with session 2 and follow-up 1 with follow-up 2 because they did not differ significantly). The results show that OBE at follow-up resulted significantly smaller than OBE prior to treatment (*t* = −2.19, *p* < 0.05, on a paired samples *t* test) and comparable to OBE in session 4.

In summary, PA was effective in reducing the bisection by walking error of patient MR. The reduction was higher for PBE than for OBE, suggesting that PA was more effective in ameliorating the initial spatial representation of the trajectory than spatial remapping during gait execution. In addition, the amelioration of neglect persisted up to 15 months post treatment.

## Discussion

As a premise, we must point out that this is a single case experimental report, and can be considered a pilot study preceding a possible larger group study. We used this design because, to our knowledge, no study exists that has addressed the issue of the prevalence of neglect patients with a dissociation between near and far space neglect in walking. Since we cannot exclude that these patients have a low incidence, we wanted to assess whether PA would work on this specific patient. When single case research designs are employed, the patient undergoes different treatments in a pseudo-randomized order, and thus acts as his/her own control (Brossart et al., 2008; Bulté and Onghena, 2008).

The aim of the present study was to investigate the effect of PA on two different bisection tasks (bisection by reaching/pointing and bisection by walking) in a right brain-damaged patient (MR) with a dissociation between near and far space neglect. In particular, MR presented with more severe neglect in far than in near space in both bisection tasks. The dissociation was stronger in the bisection by reaching/pointing than in the bisection by walking task. It is worth noting that patient MR had *left* neglect in conventional cancelation and drawing tasks, but bisected line segments to the *left* of the objective midpoint (right neglect) both in conventional line bisection and in bisection by walking tasks.

Greater severity of far space neglect was especially evident if we consider the PBE, that is the error computed on the basis of the initial direction of the walking path (see Figure 7). However, MR partially corrected her initial walking error as she approached near space, as evidenced by the significant reduction of the bisection errors when she reached the line (OBE). This walking pattern shows that patient MR updated space representation during walking according to the degree of severity of her neglect in far vs. near space. It is very likely that MR activated the most impaired representation at the beginning of each walking path in far space and the less impaired representation while approaching the target in near space. According to our hypotheses, this indicates that the representation guiding MR’s line bisection is the last representation activated during walking, i.e., near space representation.

In patient MR we also evaluated the effect of PA in bisection by pointing and in bisection by walking tasks. In the *pointing task* the effect of PA was significant starting from session no. 4, where bisection error reduced to a value close to 0%. However, neglect reappeared in the follow-up session, which occurred 3 months after session 4. This indicates that, in the case of bisection by pointing, the positive effect of PA was not long-lasting. Conversely, in the *bisection by walking task* the effect of PA was maintained for a longer time. In particular the effect was still present 15 months after the last PA session. It is worth noting that PA primarily influenced the PBE parameter, that is the walking direction estimated at the beginning of the walking path, while the trajectory curvature *per se* did not change (see Figure 11): this is evidenced by the fact that the difference between PBE and OBE prior and after PA did not significantly change. According to our hypothesis, the reduction of PBE by PA strongly suggests the restoring of the more compromised representation, i.e., far space representation, *before* gait execution. This indicates that PA has a central effect on spatial representation, directly affecting higher level components of space representation rather than influencing lower level on-line recalibration factors. Importantly, this conclusion is confirmed by the fact that PA was carried out with prismatic lenses oriented so as to deviate MR’s visual field toward the *right* space (as it is normally done in order to reduce neglect for the left side of space), despite the fact that MR showed apparent neglect for the *right side* of space in line bisection tasks (MR misbisected segments to the left of the objective midpoint in both bisection by reaching/pointing and in bisection by walking). Because in conventional copying and cancelation tests patient MR showed left side neglect, we reasoned that MR’s leftward deviation was likely to be the consequence of compensatory strategies, a sort of leftward motor hyper correction, rather than a genuine right side neglect. Indeed, a similar behavior is known to be displayed by neglect patients that might compensate for their exogenous orienting deficit and ipsilesional deviation of the subjective midline by means of relatively intact endogenous searching processes (Bartolomeo and Chokron, 2002; Huitema et al., 2006). Therefore, we used right deviating prism to be sure that we did not change the usual rehabilitation procedure employed for left side neglect patients. The rationale was that if prism adaptation acted on on-line recalibration factors, we should have found a further deviation toward the left of the patient’s walking trajectory (i.e., a worsening of bisection performance). Instead, we observed a rightward deviation that showed an improvement of neglect. This means that the effect of PA intervenes before actual walking initiation, presumably on the higher spatial representation levels preceding movement execution and known to be affected in neglect.

It is unlikely that the observed results are influenced by fatigue effects. Despite the long duration of each experimental session (approximately 2 h), the patient’s performance did not decrease with time. Indeed, if this was the case, a worse performance should be expected toward the end of the experiment. This did not happen: immediately after PA-applied during the second half of the experimental session – neglect ameliorated, as shown by the reduction of bisection errors.

A novel finding of our research is that PA obtained through manual pointing (requiring visuo-motor coordination of the upper limb) transfers to gait (requiring motor coordination of the lower limbs). In line with our results, Tilikete et al. (2001) showed that PA can extend to body regions different from the one which has been adapted and that a brief adaptation to rightward shifting prisms in a reaching task generalizes to the postural system and improves neglect patient’s postural imbalance. More recently, Savin and Morton (2008) showed that arm pointing adaptation generalizes to leg pointing (see also Morton and Bastian, 2003, for somehow different results: the authors found that PA during walking generalized to reaching, but adaptation during reaching did not generalize to walking. It is worth noting that one factor that could account for the difference between these findings and ours is that Morton and Bastian tested normal subjects: it is well known that the effect of PA on normal subjects is limited if compared with the effect on neglect patients (Colent et al., 2000; Michel et al., 2003a,b). Furthermore, direct comparison of our single case experimental results with those from small group studies should be considered with caution given the difference in the two experimental designs.

Our findings may have important implications for the rehabilitation of neglect patients. Neglect symptoms may, at least partially, spontaneously recover in the acute phase post stroke (see Farnè et al., 2004), but only a very small percentage of patients (9% in the study by Farné et al.) show a complete remission of all symptoms (Hier et al., 1983; Samuelsson et al., 1997; Katz et al., 1999). Among the symptoms that may become chronic are gait deficits that prevent neglect patients to navigate safely through the environment. Symptomatology can vary from frequent falls (Webster et al., 1995) and bumping into objects located in left space (Grossi et al., 2001) to generic locomotion problems in daily living transfer activities (Nijboer et al., 2013) and a deviated walking trajectory (Brain, 1941; Berti et al., 2002; Huitema et al., 2006).

The efforts to rehabilitate unilateral neglect are further complicated by the presence of anosognosia (Halligan and Marshall, 1998), leading to a scarce cooperation of the patient in the rehabilitation programs. As a result, the presence of neglect after stroke remains one of the major factors associated with a poor functional outcome (Denes et al., 1982; Edmans et al., 1991; Jehkonen et al., 2000). Not surprisingly, a large variety of different rehabilitation techniques have been developed in order to treat neglect (see Luauté et al., 2006 for a review). PA has demonstrated to be one of the most effective. Among the symptoms showing improvement following PA are the following: the deficit of exploration of contralesional visual-space (Ferber et al., 2003), contralesional somatosensory perception (McIntosh et al., 2002; Maravita et al., 2003; Dijkerman et al., 2004), wheel-chair navigation (Jacquin-Courtois et al., 2008), and postural imbalance (Tilikete et al., 2001; Michel et al., 2003b). To the best of our knowledge PA has never been used to correct the deviated walking trajectories of neglect patients, except for a study by Keane et al. (2006) in which two ambulatory patients were shown to improve their walking abilities after PA. However, patients in this study were simply required to walk through a hallway and the authors only reported that their walking path, directed toward the right half of the hallway prior to PA, occupied the middle of it following PA. It is therefore unclear to what extent walking profited from PA and if prisms acted at the level of gait representation, gait execution, or both. Our results show that PA acts more upon the spatial representation activated at the beginning of the walking path (when the direction of the walking trajectory is first computed) than on the modulation of spatial representation during gait execution. This result may be specific for our patient, in which the far space representation – activated at the beginning of walking – was significantly more compromised than the near space representation – activated only successively, at some point during the patient’s approach to the target. Therefore, it is not surprising that the effect of PA, in this case, is stronger on the far than on the near space representation.

A further interesting and promising characteristic of PA is the long-lasting duration of its beneficial effect upon spatial representation of gait trajectory. In our case, four sessions of PA were sufficient to produce a positive outcome which lasted up to 15 months after treatment (the duration of the effect of PA on the bisection error by reaching was smaller: 3 months after treatment the bisection error reappeared). Indeed, long-lasting effects of PA after *repeated* and *prolonged* sessions of treatment have been demonstrated in previous works. In a study by Frassinetti et al. (2002) seven neglect patients were treated with two sessions of PA per day for 2 weeks and six out of seven showed an improvement of the symptomatology, in a standardized battery of visuo-spatial tests, that was maintained up to 5 weeks after treatment. In a more recent study by Serino et al. (2007), 16 neglect patients were submitted to a PA treatment for 10 daily sessions over a period of 2 weeks and showed ameliorated visuo-spatial abilities up to 3 months after treatment. Rusconi and Carelli (2012) have shown an amelioration of neglect in seven patients after 2 weeks of treatment with PA that was maintained up to 30 months after the end of treatment. In our study, four sessions of PA distanced in time one from the other, have determined a long-lasting amelioration in MR’s neglect walking trajectory. One could argue that her improvement could alternatively be attributed to spontaneous recovery. However, as we assessed MR’s neglect 10 months after stroke, this is unlikely to be the case: it has been demonstrated that neglect symptoms tend to improve up to 6–9 months from lesion, and stabilize or get worse after such time interval (Cherney and Halper, 2001).

An interesting aspect of PA in our patient is that its positive effects increase over time: both in manual and in walking bisection tasks, pre-adaptation bisection errors in the last PA session (session 4: 67 days apart from session 1) are significantly smaller than pre-adaptation errors in the preceding PA sessions (see Figures 9 and 12) (see Fortis et al., 2010, for a similar result). Additionally, the improvement in the walking trajectory is maintained 15 month after treatment, longer than the improvement in the bisection by pointing task (McIntosh et al., 2002; Pisella et al., 2002). These results should not surprise us if we consider the important role of the cerebellum in walking and in PA. On the one hand, it is well known that cerebellar lesions can produce a gait deficit known as cerebellar gait ataxia and that the cerebellum participates in postural balance (Tilikete et al., 2001), locomotion balance and, to a lesser degree, in leg coordination (Morton and Bastian, 2003); on the other hand, lesions of the right cerebellum impair adaptation to right-shifting prisms (Pisella et al., 2005) and cerebellar activation during PA in neglect patients covariates positively with the left spatial neglect improvement (Luauté et al., 2006). Hence, the cerebellum is a good candidate to play an important role in mediating the long term improvement of walking trajectory induced by PA in our patient (indeed, MR’s lesion spared the cerebellum). To investigate this possibility, fMRI research should examine the long-term plastic changes in the cerebellum in response to PA in neglect patients with gait deficits.

An alternative (or additional) explanation of the difference in the duration of the improvement induced by PA in the two bisection tasks calls into play the role of the dorsal stream in visuo-spatial processes. Specifically, according to recent anatomical and functional animal and human data (Kravitz et al., 2011), the dorsal stream gives rise to three distinct pathways: a parieto-prefrontal pathway, a parieto-premotor pathway and a parieto-medial temporal pathway, each supporting different visuo-spatial functions. The parieto-medial temporal pathway, the retrosplenial cortex in particular, seems to be implicated in spatial-navigation. Interestingly, the retrosplenial cortex and the medial occipital-parietal cortex, which sends feedback signals to the former, are spared by the lesion in our patient; instead, the involvement of the temporal pole may have more severely affected spatial representation processes not specifically related to spatial-navigation ability, such as those implicated in the bisection by pointing task. This may be one reason why the effect of PA was more durable in the bisection by walking than in the bisection by pointing task.

In conclusion, our results show, for the first time, a long-lasting rehabilitative effect of PA on walking trajectory in a patient with chronic neglect: as few as four sessions of PA ameliorated neglect during walking for as long as 15 months post treatment. Following PA, far space neglect was reduced in our patient, allowing a better representation of gait trajectory right from the first step. Instead, the curvature of the walking trajectory did not change following PA, suggesting that PA did not influence the low level processes subserving gait execution. These results show that PA acts on high level spatial cognition rather than on peripheral sensory-motor processing and is responsible for the realignment of the egocentric frame of reference guiding our patient’s gait trajectory following treatment (Fortis et al., 2010). The results of our single case experiment support a future group study on neglect patients aimed at verifying whether PA can be employed as a long-lasting rehabilitative tool in neglect patients in which gait trajectory is deviated and are prone to the adaptation effect with prismatic goggles. Finally, we hypothesize that the cerebellum and/or the retrosplenial cortex could play a crucial role in mediating the long-lasting rehabilitative effects of PA on gait trajectory in our patient.

## Conflict of Interest Statement

The authors declare that the research was conducted in the absence of any commercial or financial relationships that could be construed as a potential conflict of interest.
